# Case of Tubercular Deposite in the Cerebellum

**Published:** 1846-10

**Authors:** P. A. Allaire

**Affiliations:** Aurora, Kane Co., Ill.


ARTICLE II.

*Case of Tubercular Deposite in the Cerebellum. Autopsical Examination.* By P. A. ALLAIRE, M. D., of Aurora, Kane Co., Ill.

S. P., a young woman aged 19, of previous good health, came under my care in February last with the following symptoms: constant and severe headache, nausea, frequent vomiting, with tenderness of epigastrium. The tongue was clean, pulse about 100, moderately full and firm; all other functions well performed. She was bled from the arm, had fomentations to the tender region, took morphine, and had the bowels moved by injection. These remedies gave much relief, and in two or three days I left her apparently convalescing from the attack.

*March 9th.*—Saw S. P. again, and found her suffering as in February. The head ache was now most intense, posteriorly; every attempt to rise in bed caused vomiting; the intellect was clear. No cause could be given for her condition, great care having been observed in diet, exercise, &c. She was treated as before, except that no bleeding was had, the neck being blistered instead; and ice-cold water constantly applied, with counter-irritation to the extremities. Considerable relief was again experienced, but the patient did not recruit her strength. The stomach remained irritable, vomiting of bilious fluid being frequent, the head ache was also at times intense in spite of remedies, while the pulse became quiet and natural. She remained much in this condition during the months of April and May. In the early part of June, a new train of symptoms became developed,—(the gastric difficulty and headache having greatly subsided, while food could be taken and digested in considerable quantities,)—temporary loss of vision, followed soon by temporary loss of hearing and speech, during these attacks, which lasted from 3 to 6 hours. The pupil was exceedingly dilated, but no other part seemed paralysed. Muscular motion and sensation remained usually good. These temporary attacks of partial paralysis continued to the termination of the case, occurring from two to three times per week at first, and gradually becoming more frequent, one or even two a day being common during the last few days. The treatment was not active during this period, but rather palliative. The manipulations of Animal Magnetism seemed very much to soothe a peculiar restlessness observed during her loss of speech, hearing, and sight; it also relieved, (as she called it) "a strange feeling of stiffness and pain" in the muscles at the back of the neck. The menstrual function continued throughout, with some diminution as to quantity, and the mental faculties seemed clear when able to speak. She sank on the 13th of July, apparently from exhaustion, without coma or convulsions.

*Examination* 12 *hours after death.* By Dr. Eastman.—The contents of the thoracic and abdominal cavities were carefully examined, but no trace of disease was found, except some slight streaks of congestion in the mucous membrane of the stomach. The skull-cap was then removed. The membranes and cerebrum were healthy throughout. About four ounces of effusion were found in the ventricles. The right lobe of the cerebellum was natural, but in the left lobe near its centre, was found a tumor 1½ inches in length, 4¼ inches in greatest circumference, and weighing five drachms. When cut into, it presented much the appearance of solid tubercle, but rather firmer, except in its centre, where it was nearly soft. The tumor was not enclosed in a cyst, but seemed in immediate contact with a slightly softened layer of cerebral substance. No vessels were found going to it, nor did it seem to have any real connexion with surrounding parts, but could be turned out of its cavity as a pea-nut out of its shell. The origins of the nerves looked well, with the exception of those of the optic, which were slightly wasted.

The point most worthy of note in the above detail, seems to be the marked sympathetic affection which existed in the stomach and liver, particularly in the stomach, during the first three or four months of the disease. This was so great as to call at first a large share of attention and treatment to a part in which existed no real change, and which in fact maintained its integrity to the last. On this account the case seems to show the importance of a more correct knowledge of the various sympathetic affections than has yet been attained, particularly as they throw so much light, when understood, on the etiology of diseases, their seat, and the organ to which our therapeutic means should be directed.

July 18, 1846.

